# Genetic Pharmacotherapy as an Early CNS Drug Development Strategy: Testing Glutaminase Inhibition for Schizophrenia Treatment in Adult Mice

**DOI:** 10.3389/fnsys.2015.00165

**Published:** 2016-01-08

**Authors:** Susana Mingote, Justine Masson, Celia Gellman, Gretchen M. Thomsen, Chyuan-Sheng Lin, Robert J. Merker, Inna Gaisler-Salomon, Yvonne Wang, Rachel Ernst, René Hen, Stephen Rayport

**Affiliations:** ^1^Department of Psychiatry, Columbia UniversityNew York, NY, USA; ^2^Department of Molecular Therapeutics, New York State Psychiatric InstituteNew York, NY, USA; ^3^Centre de Psychiatrie et Neurosciences, Institut National de la Santé et de la Recherche Médicale UMR 894 and Université Paris DescartesParis, France; ^4^Department of Pathology and Cell Biology, Columbia UniversityNew York, NY, USA; ^5^Department of Integrative Neuroscience, New York State Psychiatric InstituteNew York, NY, USA; ^6^Psychobiology Labs, Department of Psychology, University of HaifaHaifa, Israel; ^7^Departments of Neuroscience and Pharmacology, Columbia UniversityNew York, NY, USA

**Keywords:** glutamate, glutamine, GLS1, tamoxifen-inducible, allelic abundance, antipsychotic

## Abstract

Genetic pharmacotherapy is an early drug development strategy for the identification of novel CNS targets in mouse models prior to the development of specific ligands. Here for the first time, we have implemented this strategy to address the potential therapeutic value of a glutamate-based pharmacotherapy for schizophrenia involving inhibition of the glutamate recycling enzyme phosphate-activated glutaminase. Mice constitutively heterozygous for GLS1, the gene encoding glutaminase, manifest a schizophrenia resilience phenotype, a key dimension of which is an attenuated locomotor response to propsychotic amphetamine challenge. If resilience is due to glutaminase deficiency in adulthood, then glutaminase inhibitors should have therapeutic potential. However, this has been difficult to test given the dearth of neuroactive glutaminase inhibitors. So, we used genetic pharmacotherapy to ask whether adult induction of GLS1 heterozygosity would attenuate amphetamine responsiveness. We generated conditional floxGLS1 mice and crossed them with global CAG^ERT2*cre*∕+^ mice to produce GLS1 iHET mice, susceptible to tamoxifen induction of GLS1 heterozygosity. One month after tamoxifen treatment of adult GLS1 iHET mice, we found a 50% reduction in GLS1 allelic abundance and glutaminase mRNA levels in the brain. While GLS1 iHET mice showed some recombination prior to tamoxifen, there was no impact on mRNA levels. We then asked whether induction of GLS heterozygosity would attenuate the locomotor response to propsychotic amphetamine challenge. Before tamoxifen, control and GLS1 iHET mice did not differ in their response to amphetamine. One month after tamoxifen treatment, amphetamine-induced hyperlocomotion was blocked in GLS1 iHET mice. The block was largely maintained after 5 months. Thus, a genetically induced glutaminase reduction—mimicking pharmacological inhibition—strongly attenuated the response to a propsychotic challenge, suggesting that glutaminase may be a novel target for the pharmacotherapy of schizophrenia. These results demonstrate how genetic pharmacotherapy can be implemented to test a CNS target in advance of the development of specific neuroactive inhibitors. We discuss further the advantages, limitations, and feasibility of the wider application of genetic pharmacotherapy for neuropsychiatric drug development.

## Introduction

Genetic pharmacotherapy—the use of genetic intervention to achieve a pharmacological effect—has been proposed as an early drug development strategy for the identification of novel CNS targets in mouse models prior to the development of specific ligands (Gellman et al., 2011). This strategy offers particular advantages for proof-of-concept studies for CNS disorders. Currently, identifying drug candidates for CNS disorders requires not only designing specific high-affinity ligands, with minimal off-target effects, but also that the ligands permeate the blood-brain barrier. Genetic pharmacotherapy obviates these ligand-development requirements by utilizing tamoxifen as a wild-card ligand, with excellent brain permeation, and achieves perfect specificity through genetically controlled knockdown of the gene of interest.

Genetic pharmacotherapy employs ubiquitous and temporally controlled genetic blockade to simulate systemic drug treatment in adulthood. Both the global and temporal aspects of this strategy can be achieved by breeding a global tamoxifen-inducible cre mouse line, in which ERT2*cre* is under the control of a strong ubiquitous promoter, with a mouse line in which the gene encoding the molecular target of interest is floxed. Utilizing heterozygous floxed mice enables end-stopping the induced deficiency at about 50%, matching the range of inhibition achieved by most drugs used in the treatment of psychiatric disorders (Farde et al., 1992; Hirano et al., 2005). Despite the apparent advantages of the genetic pharmacotherapy strategy, its success in uncovering new targets for the treatment of psychiatric disorders has not been tested.

Identifying novel therapeutic targets for the treatment of schizophrenia (SCZ) has been particularly challenging. Despite the increasing impetus for glutamate-based pharmacotherapies for SCZ, none have yet proven successful (Moghaddam and Javitt, 2012). Plausible explanations are that current glutamatergic pharmacotherapeutic targets do not achieve the necessary modulation of aberrant synaptic activity or do not target key brain circuits selectively. Targeting glutamate synaptic transmission presynaptically has therapeutic potential (Conn et al., 2009). Metabotropic mGluR2/3 agonists attenuate both PCP-induced glutamate release and PCP-induced psychomotor stimulation (Moghaddam and Javitt, 2012). This preclinical work culminated in the demonstration of significant clinical promise for the mGluR2/3 agonist LY214002 in early clinical trials (Patil et al., 2007); however, this was not borne out in subsequent studies (Adams et al., 2014; Downing et al., 2014), although subtype selective modulation holds considerable promise (Walker and Conn, 2015).

An alternate presynaptic glutamate-based approach involves inhibiting glutamate recycling. Metabolic studies indicate that the majority of synaptically released glutamate is synthesized or recycled from glutamine via the action of glutaminase (Albrecht et al., 2010; Rothman et al., 2011), and electrophysiological studies indicate that excitatory synaptic transmission can be attenuated by inhibition of glutaminase (Tani et al., 2014). Consistent with this, homozygous stopGLS1 mice (GLS1 knockout mice) die shortly after birth, apparently due to altered rhythmic activity in respiratory centers (Masson et al., 2006). In culture, homozygous stopGLS1 neurons show normal spontaneous excitatory synaptic activity, but more pronounced synaptic fatigue when stimulated at higher frequency, consistent with the glutamate recycling function of glutaminase. In adult hippocampal slices, excitatory transmission is modulated by reducing or enhancing glutamine, dependent on time (Kam and Nicoll, 2007) and patterns of synaptic activity (Tani et al., 2014). Thus, glutaminase inhibition is likely to attenuate high-frequency excitatory activity preferentially.

Examining mouse models with resilience—rather than disease—phenotypes offers a more direct approach to identifying therapeutic targets for complex neuropsychiatric disorders (Mihali et al., 2012). Remarkably, heterozygous stopGLS1 mice (GLS1 HETs), with only one functional GLS1 allele, manifest a SCZ resilience phenotype (Gaisler-Salomon et al., 2009a), with diminished responsiveness to propsychotic amphetamine challenge and reduced amphetamine-induced dopamine release. On brain imaging, GLS1 HET mice show hypoactivity in hippocampal CA1, inverse to that seen in the clinical studies, as well as attenuated ketamine-induced frontal cortex activation (Gaisler-Salomon et al., 2009b). Taken together these findings suggest that systemic administration of glutaminase inhibitors might prove therapeutic in SCZ. Importantly, partial inhibition of glutaminase appears to have a benign side-effect profile, as GLS1 HETs are remarkably normal in a wide-ranging battery of behavioral tests of baseline behavior (Gaisler-Salomon et al., 2009a). They do have a subtle cognitive phenotype, with a reduction in delayed context-dependent fear conditioning (Gaisler-Salomon et al., 2009a), with adult onset (Gaisler-Salomon et al., 2012), and an enhancement in trace fear conditioning (Hazan and Gaisler-Salomon, 2014). The lack of high potency brain-penetrant glutaminase inhibitors has precluded testing glutaminase inhibition as a pharmacotherapy for SCZ.

Here we have implemented a genetic pharmacotherapy strategy for the first time in the CNS to ask whether reducing GLS1 expression to heterozygous levels in adult mice would block the behavioral response to propsychotic amphetamine challenge. There are three steps in the strategy. In the first step, we made floxGLS1 mice, in which exon 1 of GLS is susceptible to cre-dependent recombination to reduce GLS1 expression, and bred these mice with global inducible deletor CAG^ERT2*cre*^ mice, in which the CAG promoter drives tamoxifen-inducible cre expression to enable pharmacological inhibition to about 50%. In the second step, we show in the resulting progeny that tamoxifen (Tmx) induces full recombination of the floxGLS1 allele, and reduction in GLS1 expression to about 50%. In the third step, we ask whether the induced GLS1 reduction attenuates amphetamine-induced hyperlocomotion.

## Materials and methods

### Mice

Procedures involving mice and their care were conducted in accordance with the guidelines of the National Institutes of Health *Guide for the Care and Use of Laboratory Animals*, under protocols approved by the Institutional Animal Care and Use Committees of Columbia University and New York State Psychiatric Institute. Matched littermates were used as controls. Experimental mice were 50:50 C57BL/6J:129J background. Both sexes were used, in roughly equal proportions for molecular studies; male mice were used for behavioral studies.

### Generation of conditional GLS1 HETs

**Figure 1:**
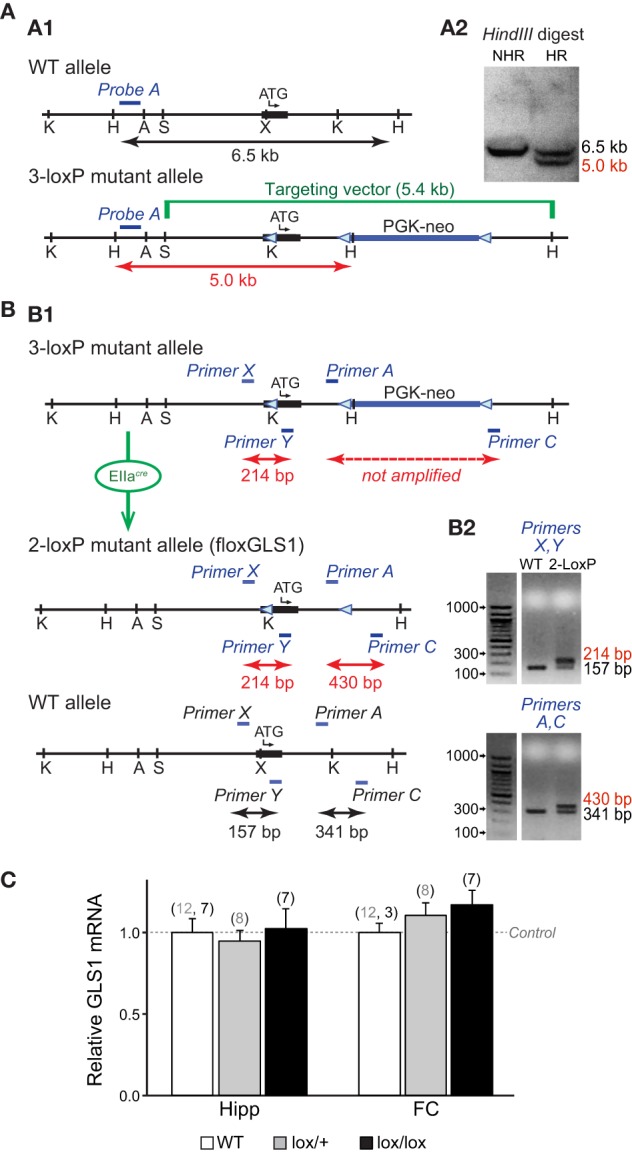
**Generation of conditional floxGLS1 mice. (A)** Insertion of 3-loxP construct into the GLS1 gene. **(A1)** A targeting construct engineered with loxP sites flanking exon 1 (thick black line with transcription start ATG codon indicated) of the GLS1 gene encoding glutaminase was inserted into the endogenous GLS1 locus by homologous recombination. The targeting vector (green) also contained a floxed PGK-neo-cassette (thick blue line) for positive selection of the ES cell clones. The blue bar indicates the position of external *Probe A* (blue), used for Southern analysis, while the black arrow indicates the length of the expected wild-type band and the red arrow the length of the expected mutant band. Note that an additional *HindIII* site was introduced along with the middle loxP site. **(A2)** In a *HindIII* digest of ES cell DNA, *Probe A* hybridized to a 6.5 kb band for the WT allele, in contrast to a 5.0 kb band for the 3-loxP allele. Results are shown for ES cells with non-homologous recombination (NHR) and homologous recombination (HR). **(B)** Breeding floxGLS1 mouse. **(B1)** 3-loxP mice were crossed with EIIa^*cre*^ partial deletor mice to remove the floxed PGK-neo cassette. 2-loxP progeny were identified by PCR genotyping using two primer pairs flanking the loxP sites. Blue bars indicate the location of the primers. **(B2)** The PCR gels show genotyping results for two mice. In the top gel, the 5′ loxP site was revealed by a 214 bp band, in addition to the WT allele of 157 bp in the mutant mouse (2-loxP). In the bottom gel, the 3′ loxP site was revealed by a 430 bp band, in addition to the WT and of 341 bp in the mutant mouse. **(C)** GLS1 mRNA expression from the floxGLS1 allele. mRNA was measured in whole hippocampus (Hipp) and frontal cortex (FC) tissue. Relative expression was normalized to the corresponding WT mice (dashed line), done in two cohorts of mice, *lox*/+ and *lox/lox* mice. There was no genotypic impact of GLS1 flox status on GLS1 mRNA. The number of samples is indicated above the bars (gray numbers correspond to the *lox*/+ cohort and black numbers correspond the *lox/lox* cohort).

Conditional GLS1 mice were made by introducing one-loxP site into exon 1 and a floxed PGK-neo cassette into intron 1 (Figure 1A1). The *SphI*-*HindIII* targeting vector of 5.4 kb inserted in exon 1 contained a loxP site 16 bp before the initiating codon and a floxed 1018 bp neomycin-resistance (neo) cassette in intron 1. The three-loxP sites were in reverse orientation. The loxP site before the ATG was inserted into a unique *XhoI* site that was created by site-directed mutagenesis. A *KpnI* site was added at the 5′ end of the oligonucleotide coding for the loxP site, and a *HindIII* site was added in the floxed PGK-neo cassette to facilitate subsequent screening by Southern analysis, after electroporation of the targeting vector into embryonic stem (ES) cells. Southern blot analysis used a ^32^P-labeled external probe (Probe A: 336 bp genomic probe 5′ of the targeting construct; Figure 1A2).

Chimeric 3-loxP mice resulting from the implantation of selected ES cell clones were bred to wild-type (WT) mice for one generation and then crossed to EIIa^*cre*^ universal partial deletor mice (Holzenberger et al., 2000); F1 progeny (mosaic mice) were bred with wild-type mice (Taconic, strain 129JS6/SvEvTac) to obtain progeny with the 3-loxP allele, the 2-loxP allele, or 1-loxP (delta allele; Figure 1B1). F2 progeny with the desired 2-loxP allele were identified by PCR genotyping. The 5′ loxP site was identified by forward primer (*Primer X*) GCCCCAAGCATCCTCATCTCGAATA and reverse primer (*Primer Y*) TAAGAGCAGCTCCCGTAGCA; the 3′ loxP site was identified by forward primer (*Primer A*) GGCCTGCTTAATGTTTCCTG and reverse primer (*Primer C*) GGCATATCCCTGAGTTCGAG (Figure 1B2). GLS1^*lox*∕+^ mice were bred to generate GLS1^*lox*∕+^ and GLS1^*lox*∕*lox*^ mice for assessment of genotypic impact of flox status on GLS1 mRNA expression.

### Generation of inducible GLS1 heterozygous mice

We used Tmx-inducible CAG^ERT2*cre*^ (Hayashi, 2002) mice obtained from The Jackson Laboratory [JAX; strain B6.Cg-Tg(Cag-cre/Esr1^*^)5Amc/J; Stock number 004682]. CAG^ERT2*cre*∕+^ mice were first bred with a enhanced yellow fluorescence (EYFP) reporter mouse Rosa26^*sf*EYFP∕+^[JAX, B6.129X1-*Gt(ROSA)26Sortm1(EYFP)Cos*/J; stock number 006148] to confirm ubiquitous *cre* expression. The genotyping of these mice was done following the JAX protocol and primers. The CAG^ERT2*cre*∕+^ mice were then crossed with GLS1^*lox*∕*lox*^ mice, to obtain experimental GLS1 iHET and control mice. For genotyping, we used the previously described forward *Primer A* and reverse *Primer C* for the floxed allele and *Primer X* and reverse *Primer C* for the delta allele.

### RNA extraction and reverse transcription quantitative PCR (RT-qPCR)

Brain tissue was flash-frozen in 300 μl of Qiazol (Qiagen), a RNase-inhibitor buffer. RNA extraction was done using the RNeasy Lipid Mini Kit (Qiagen), according to the manufacturer's instructions and stored in RNase-free water at −80°C. RT-qPCR was performed as described previously (Gaisler-Salomon et al., 2012). Briefly, RNA concentrations were standardized to 1 μg per 10 μl water using a NanoDrop 1000 Spectrophotometer (ThermoScientific, Waltham, MA). The 260:280 nm absorbance ratio was measured to assess RNA quality; samples were excluded if the ratio was outside the range 2.0 ± 0.2, or if the RNA concentration was too low. Genomic DNA was removed by digestion with 1 μl DNase (Promega). Reverse transcription was carried out using a High Capacity cDNA Reverse Transcription kit (Applied Biosystems). Reverse transcription product (cDNA) was diluted to a volume of 1 ml in water. qPCR was performed using an Opticon 2 DNA Engine (Bio-Rad) and GPDH primers (forward: AACTCCCAC TCTTCCACCT; reverse: CACCACCCTGTTGCTGTA) and GLS1 primers (forward: GTACAGTCTCTGTGGCTTGG; reverse: CAGTTAGCGGCTCATTCA C). The cycle threshold (Ct) values for GLS1 gene were normalized to GAPDH (ΔCt). Relative copy number was obtained by exponentiation of ΔCt values (function 2^−ΔCT^)

### Tamoxifen administration

A 10 mg/ml Tmx solution was prepared by solubilizing 125 mg tamoxifen (Sigma-Aldrich, T5648) in 1.25 ml 100% ethanol and then 11.25 ml peanut oil (Sigma-Aldrich, P2144). The mixture was vortexed for 5 min and incubated for >6 h at 37°C. Animals received 0.1 mL i.p. (1 mg Tmx) daily for 5 successive days.

### Immunohistochemical visualization of EYFP expression

Mice (Rosa26^*fs*EYFP∕+^:: CAG^ERT2*cre*∕+^ or Rosa26^*fs*EYFP∕+^) were perfused with cold PBS followed by 4% paraformaldehyde (PFA), the brains removed, post-fixed overnight in 4% PFA, and cryoprotected in 30% sucrose. Brains were then cut using a cryostat. Sections (50 μm) were washed in PBS (100 mM; pH 7.4) and incubated in glycine (100 mM) for 30 min to quench aldehydes. Non-specific binding was blocked with 10% normal goat serum (NGS; Millipore) in 0.1% PBS Triton X-100 for 2 h (PBS-T). Sections were incubated with primary antibody, anti-GFP (1:2000 dilution; rabbit polyclonal antibody; Millipore, AB3080) in 0.02% PBS-T and 2% NGS at 4°C for 24 h, then washed with PBS and incubated for 45 min with anti-rabbit Alexa Fluor 488 (1:200 dilution; Invitrogen) in 0.02% PBS-T. Sections were mounted on slides and cover slipped using Prolong Gold aqueous medium (Invitrogen) and stored at 4°C. Fluorescence images were acquired with a Fluoview FV1000 confocal laser scanning microscope (Olympus) with a 20x objective.

### Quantitative GLS1 genotyping

The left hippocampus of Control and GLS1 iHET mice was used for quantitative genotyping (and the right for RNA expression). Tissue was sent to Transnetyx (Cordova, TN) in 96-well plates for genotyping using probe-based quantitative PCR (qPCR). Allelic abundance was obtained from the mean of 4 qPCR determinations (2 runs done in duplicate). The conditional floxGLS1 and wild-type allele signals were normalized to the one-allele signal from control (GLS1^lox∕+^) mice that had not received Tmx.

### Amphetamine-induced hyperlocomotion

Control and GLS1 iHET mice were handled for 5 min daily for 3 days prior to amphetamine challenge. Locomotor activity and rearing was measured using SmartFrame Open Field Stations (Kinder Scientific) equipped with infrared beams at 2 different heights; each break of a lower beam represents an ambulatory count, while each break of a upper beam represents a rearing count. We also measured fine movements, defined as beam breaks that do not reflect the relocation of the mouse position in the open field, and that are sensitive to movements like grooming and head weaves or bobs. On the test day, mice were put in the open field for 1 h, then administered amphetamine 2 mg/kg via intraperitoneal injection and returned immediately to the open field, and monitored for the subsequent 2 h. D-Amphetamine hemisulfate (Sigma-Aldrich) was dissolved in normal saline.

### Statistical analysis

The data for allelic abundance and mRNA expression were analyzed using a genotype x Tmx treatment factorial ANOVA. Significant interactions were analyzed further for simple main effects using the error terms from the specific analyses. The percentage of control mRNA values in the hippocampus were analyzed using a one-way ANOVA followed by the LSD *post-hoc* test. The percentage of control mRNA values for the Tmx-Treated groups were analyzed using a brain region x time (post-Tmx injection) ANOVA. Behavioral data recorded in bins of 10 min during the 3 h open field session were analyzed separately for each Tmx treatment using factorial ANOVA with genotype as the between-subjects factor and time (bin) as the within-subjects repeated measures factor. Total locomotion in response to amphetamine was used to compare between treatments (No-Tmx and Tmx-Treated, at the 30 day time point) using genotype × treatment factorial ANOVA. Significant interactions were further analyzed for simple main effects using the error terms from the specific analyses. Total locomotion in response to amphetamine of Tmx-Treated mice at the 30 and 146 day time points was analyzed using a repeated measures ANOVA.

## Results

### Conditional floxGLS1 mice

We generated conditional floxGLS1 mice by knocking in a 3-loxP cassette (Targeting vector), with lox P sites flanking the coding sequence of GLS1 exon 1, followed by a floxed PGK-neo cassette (Figure 1A1). ES cells with successful recombination events were identified by Southern analysis of a HindIII restriction digest. An external probe, *Probe A*, identified a band of genomic DNA of 5 kb in ES cells showing homologous recombination, in contrast to a band of 6.5 kb in WT ES cells (Figure 1A2). Southern analysis of a KpnI restriction digest with *Probe A* identified a band of 4.9 kb in ES cells showing homologous recombination, in contrast to a band of 7.2 kb in WT ES cells (data not shown). The PGK-neo cassette was removed by breeding 3-loxP mice with EIIa^*cre*^ universal partial deletor mice (Holzenberger et al., 2000) and genotyping the progeny for the desired 2-loxP floxGLS1 allele (Figure 1B1). The 5′ loxP site was identified with *Primers X* and *Y*, yielding a mutant band containing the loxP site of 214 bp, in contrast to a WT band of 157 bp. The 3′ loxP site was identified with *Primers A* and *C*, yielding a mutant band containing the loxP site of 430 bp, in contrast to a WT band of 341 bp (Figure 1B2). Analysis of GLS1 mRNA revealed no genotypic impact of flox status (Figure 1C), in both the hippocampus [*F*_(2, 31)_ = 0.08, *p* = 0.93] and frontal cortex [*F*_(2, 27)_ = 0.68, *p* = 0.51].

**Figure 2:**
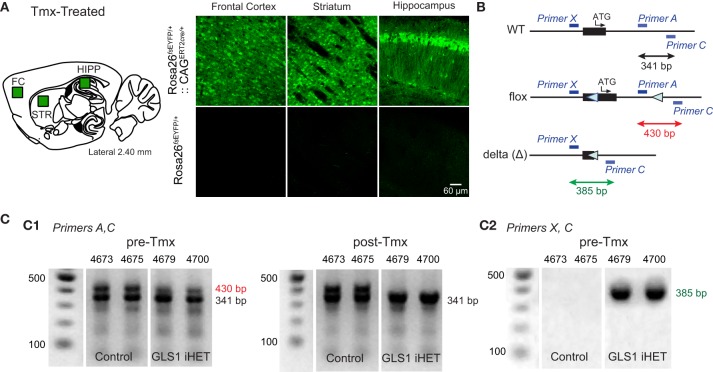
**Tamoxifen induction of GLS1 deficiency. (A)** Test for universal cre action. CAG^ERT2*cre*∕+^:: Rosa26^*fs*EYFP∕+^ mice and control Rosa26^*fs*EYFP∕+^ mice were treated with Tmx, and their brains subsequently immunostained to visualize EYFP, in the frontal cortex, striatum and hippocampus, as indicated on the sagittal brain section schematic (*left*). Confocal images revealed ubiquitous EYFP expression in CAG^ERT2*cre*∕+^:: Rosa26^*fs*EYFP∕+^, but not Rosa26^*fs*EYFP∕+^ mice. **(B)** Primer strategy used to determine the presence of WT, floxed, and recombined (Δ) GLS1 alleles. Blue bars indicate the locations of the three primers used. Black arrow indicates length of the amplified WT allele, the red arrow the floxed allele and the green arrow the recombined (Δ) allele. **(C)** PCR genotyping of tail samples from control and GLS1 iHET mice pre- and post-Tmx. Gels from two mice of each genotype are shown, with mouse identification numbers. Band sizes are shown to the right of the gels. **(C1)** Gel electrophoresis (*left*) showed both floxed and WT DNA bands (with *Primers A,C*) prior to Tmx (Pre-Tmx) administration in GLS1 iHET and Control mice, whereas post-Tmx (*right*), the floxed DNA band remained in Control (GLS1^*lox*∕+^) mice, but was eliminated in GLS1 iHET mice. **(C2)** Gel electrophoresis with *Primers X, C* identified the presence of a Δ allele in GLS1 iHET mice prior to Tmx treatment, revealing “leaky” cre-mediated recombination.

To enable Tmx-induced recombination of the floxGLS1 allele, we used CAG^ERT2*cre*^ global-inducible deletor mice. To demonstrate universal *cre* induction, we first bred the mice with Rosa26^*fs*EYFP^ EYFP reporter mice. After Tmx, Rosa26^*fs*EYFP∕+^:: CAG^ERT2*cre*∕+^, but not Rosa26 ^*fs*EYFP∕+^, mice showed widespread EYFP expression in all brain areas imaged (Figure 2A). Then, we crossed CAG^ERT2*cre*∕+^ mice with GLS1^*lox*∕*lox*^ mice to produce experimental GLS1 inducible-Het (GLS1 iHET), CAG^ERT2*cre*∕+^:: GLS1^lox∕+^ mice and Control GLS1^*lox*∕+^ mice. We used PCR screening to detect the presence of WT, floxed and recombined (Δ) alleles (Figure 2B). The PCR gels of tail samples showed that prior to Tmx, WT and floxed allele bands were present in both Control (*n* = 5) and GLS1 iHET (*n* = 8) mice (Figure 2C1, *left*). After Tmx, PCR gels from GLS1 iHET mice showed complete elimination of the floxed allele band, while PCR gels from control mice continued to show both the WT and floxed allele bands (Figure 2C1, *right*).

To ask whether there was recombination prior to Tmx, we looked for the presence of the Δ allele band in tails samples (Figure 2C2), identified as a 390 bp band with Primers *X* and *C*. Although the Δ band was not present in control mice, there was a prominent Δ band in Tmx-naïve GLS1 iHET mice. Since PCR gels are not quantitative, this revealed only that there was some “leaky” *cre* recombination prior to Tmx administration in tail samples, but did not provide quantitative information about the magnitude of the leakiness.

### Conditional GLS1 reduction in the brain

**Figure 3:**
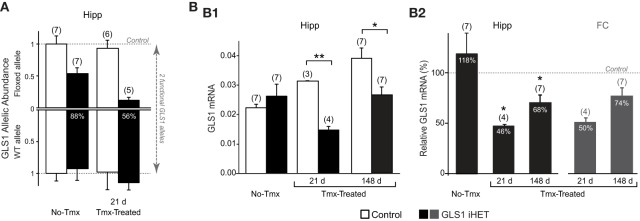
**Induction of GLS1 deficiency in the brain. (A)** GLS1 allelic abundance. Wild type (WT) and floxGLS1 (Floxed) allelic abundance in one hippocampus is plotted with respect to the allelic abundance in Control, Tmx-naïve (No-Tmx) mice. In Tmx-naïve GLS1 iHET mice, the floxed allele abundance was 0.54, while in Tmx-treated GLS1 iHET mice this went down to 0.12. The percent reduction in functional allelic abundance is indicated on the bars. **(B)** Relative GLS1 mRNA expression. **(B1)** In Tmx-naïve mice (No-Tmx), there was no genotypic difference in GLS1 mRNA expression in the hippocampus, while in Tmx-treated mice, there was a significant main effect of genotype at both time points after Tmx injection (21 days and 148 days). ^**^indicates *p* < 0.001 ^*^indicates *p* < 0.05. **(B2)** mRNA values for the hippocampus (Hipp) in GLS1 iHET mice were expressed as the percentage of control values for each condition (dashed line). There was no reduction in Tmx-naïve mice, whereas in Tmx-treated mice relative mRNA expression was reduced at both 21 and 148 days post-Tmx. ^*^statistically different from No-Tmx GLS1 iHET mice, *p* < 0.05. The relative mRNA values for the frontal cortex (FC) of Tmx-treated mice (dark gray bars) also showed a similar reduction, to 50 and 74%, 21 and 148 days post-Tmx, respectively.

To assess the effect of Tmx treatment in the brain of GLS1 iHET mice, we measured GLS1 allelic abundance by quantitative genotyping with RT-qPCR, in the whole hippocampus from one hemisphere of Tmx-naïve (No-Tmx) mice at P60 and Tmx-Treated-21 d mice (P81), 21 days post-Tmx.We compared No-Tmx Control mice (*n* = 7) and GLS1 iHET mice (*n* = 7) to Tmx-Treated-21 d Control (*n* = 6) and GLS1 iHET mice (*n* = 5; Figure 3A). As expected, WT allelic abundance did not change; there was no significant genotypic [*F*_(1, 21)_ = 3.98, *p* = 0.06] or Tmx treatment [*F*_(1, 21)_ = 1.13, *p* = 0.30] effect, and there were no significant interactions [*F*_(1, 21)_ = 0.77, *p* = 0.39]. Floxed GLS1 allelic abundance was lower in the GLS1 iHET mice prior to Tmx and nearly eliminated post-Tmx. Factorial ANOVA revealed a significant effect of genotype [*F*_(1, 21)_ = 42.05, *p* < 0.0001] and treatment [*F*_(1, 21)_ = 6.03, *p* = 0.02], but no significant interaction [*F*_(1, 21)_ = 3.16, *p* = 0.09], consistent with “leaky” *cre* recombination. In GLS1 iHET No-Tmx hippocampal tissue there was a 43% decrease in floxed GLS1 allelic abundance, corresponding to 77% functional GLS1 abundance. Post-Tmx, there was an 88% decrease in floxed GLS1 allelic abundance, corresponding to 56% functional GLS1 abundance.

We then measured GLS1 mRNA expression in the other hippocampi of the same mice used for the RT-qPCR allelic abundance determination, combined with hippocampi from Tmx-Treated mice used in the behavioral experiment (see below). We compared No-Tmx, Control (*n* = 7; GLS1 iHET, *n* = 7) to Tmx-Treated-21 d (Control, *n* = 3; GLS1 iHET, *n* = 4) and Tmx-Treated-148 d (Control, *n* = 7; GLS1 iHET, *n* = 7; Figure 3B1). Factorial ANOVA revealed a significant genotype x treatment interaction [*F*_(1, 28)_ = 6.61, *p* = 0.004]. Among treatment conditions, there was no genotype effect in the No-Tmx group [*F*_(1, 11)_ = 0.79, *p* = 0.39], but a significant genotype effect in the Tmx-Treated-21 d group [*F*_(1, 5)_ = 462.07, *p* < 0.0001] and the Tmx-Treated-148 d group [*F*_(1, 12)_ = 9.33, *p* = 0.01]. Despite some “leaky” recombination, these results revealed that there was no genotypic impact on GLS1 mRNA levels prior to Tmx treatment.

GLS1 mRNA increased significantly in Control mice [*F*_(1, 14)_ = 14.465, *p* < 0.001], but not in GLS1 iHET mice [treatment effect: *F*_(1, 14)_ = 3.417, *p* = 0.06]. The increase in Control mice was likely due to the different ages of the three groups: No-Tmx were P60; Tmx-Treated-21 d were P81 (21 days older), and Tmx-Treated-148 d ranged from P218–P282 (~5 months older). To avoid the age confound, we analyzed normalized ratios of GLS1 mRNA expression (Figure 3B2, right). This revealed that GLS1 mRNA expression in the hippocampus was reduced to 46% in Tmx-Treated-21 d mice and to 68% in Tmx-Treated-148 d mice. A one-way ANOVA showed a treatment effect [*F*_(2, 14)_ = 6.865, *p* = 0.008). LSD *post-hoc* revealed that mRNA expression ratios in No-Tmx mice were different from Tmx-Treated-21 d (*p* = 0.004) and Tmx-Treated-148 d mice (*p* = 0.015), but there was no statistical difference between Tmx-Treated-21 d and Tmx-Treated-148 d mice (*p* = 0.294). A similar reduction was seen in the frontal cortex, 50% in Tmx-Treated-21 d mice and 78% in Tmx-Treated-148 d mice (Figure 3B2, left), consistent with an induced global reduction in GLS1 expression to heterozygous levels. ANOVA results comparing the mRNA values between hippocampus and frontal cortex of Tmx-Treated mice at the two time points, revealed a main effect of time [*F*_(1, 18)_ = 10.47, *p* = 0.005], but not brain region [*F*_(1, 18)_ = 0.569, *p* = 0.46], nor a brain region x time interaction [*F*_(1, 18)_ = 0.024, *p* = 0.46], suggesting that after 5 months there was some recovery of function at the mRNA level.

### Amphetamine-induced hyperlocomotion

**Figure 4:**
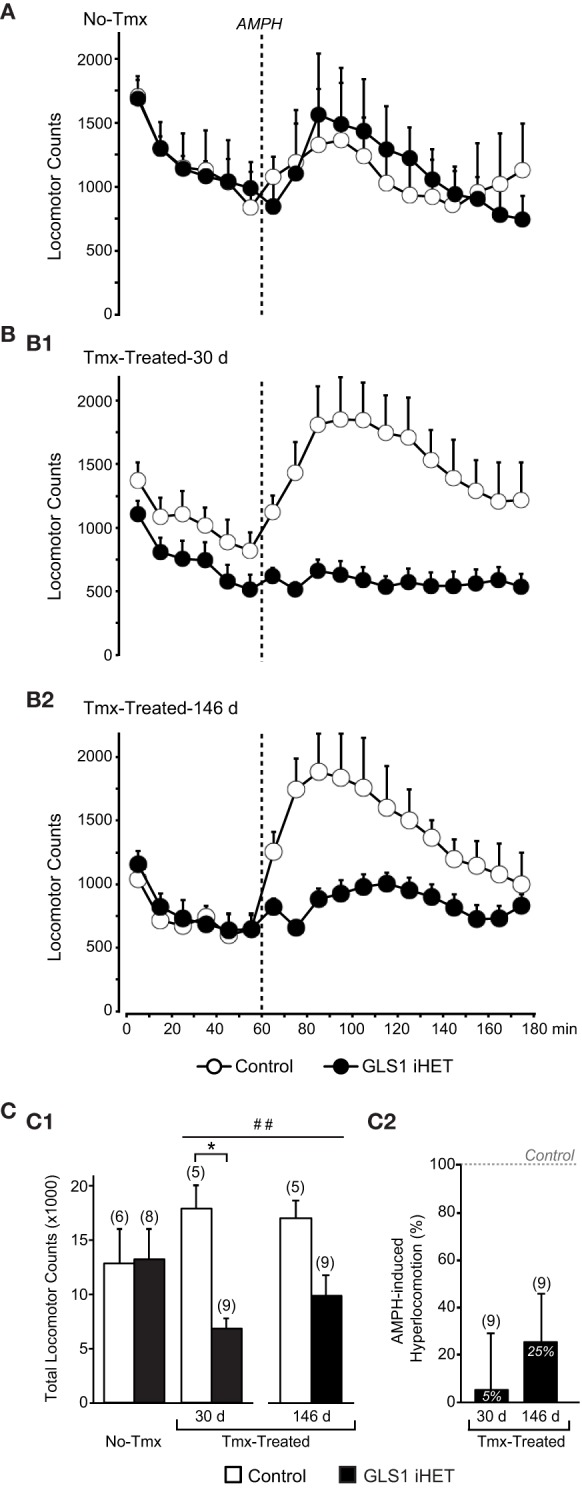
**Attenuation of amphetamine-induced hyperlocomotion in Tmx-Treated GLS1 iHet mice. (A)** Amphetamine-induced locomotion. Tmx-naïve (No-Tmx) mice prior to and after amphetamine (AMPH) injection showed no genotypic difference in locomotion in the open field. **(B)** Locomotion before and after amphetamine for Tmx-treated mice. **(B1)** Tmx-Treated mice showed a marked genotypic difference in amphetamine-induced hyperlocomotion, 30 days post-Tmx. Factorial ANOVA revealed a significant genotype × time effect of amphetamine (*p* < 0.05), but no difference prior to amphetamine. **(B2)** The reduction in amphetamine-induced hyperlocomotion persisted 146 days post-Tmx. **(C)** Total locomotion after amphetamine injection. **(C1)** Tmx-naïve (No-Tmx) mice showed no genotypic difference in their overall response to amphetamine, whereas Tmx-Treated control mice differed significantly from GLS1 iHET mice 30 days post-Tmx (left graph). ^*^indicates a significant genotypic difference, *p* < 0.05. The amphetamine-induced locomotion of Tmx-Treated mice did not change 146 days post-Tmx treatment. ^*##*^indicates a main effect of genotype, *p* < 0.001; but no main effect of time or significant interaction. **(C2)** AMPH-induced hyperlocomotion expressed as an increase in locomotion above baseline. Tmx-Treated GLS1 iHET mice showed a minimal response to amphetamine at 30 days post-Tmx (5% increase in locomotion in response to AMPH), and a modest response at 146 days (25%).

We then asked whether adult-induced glutaminase deficiency would attenuate amphetamine-induced hyperlocomotion. We compared two cohorts of mice: No-Tmx Control (*n* = 6) and GLS1 iHET mice (*n* = 8; Figure 4A), and Tmx-Treated Control (*n* = 5) and GLS1 iHET mice (*n* = 9; Figure 4B). Tmx-Treated mice (age range P74–P141) received Tmx 1 mg daily, for 5 days, and were tested 30 days after the last Tmx injection (age range P117–P174), and then again after 146 days (age range P226–P283). There was no genotypic difference in the weight of Tmx-treated mice at 30 days post-Tmx (Control, 34.78 ± 1.88 g; GLS1 iHET, 36.14 ± 1.3 g; *p* = 0.611) nor at 146 days (Control, 39.54 ± 2.89 g; GLS1 iHET, 42.31± 1.2 g; *p* = 0.283).

Mice were allowed to habituate to the open field for 1 h. Although Control Tmx-treated-30 d mice appeared to be more active during habituation than Tmx-treated-30 d GLS1 iHET mice, the difference was not significant. During habituation, there was no significant genotype × time interaction in the three groups of mice: for No-Tmx mice [ANOVA with time as the repeated measure factor, *F*_(1, 12)_ = 0.27, *p* = 0.61; Figure 4A], Tmx-Treated-30 d mice [*F*_(1, 12)_ = 0.52, *p* = 0.82; Figure 4B1], or Tmx-Treated-146 d mice [*F*_(1, 12)_ = 0.432, *p* = 0.43; Figure 4B2].

After habituation, mice received amphetamine (2 mg/kg) and were monitored for 2 h. There was no significant genotype × time interaction in No-Tmx mice [ANOVA with time as the repeated measure factor, *F*_(11, 132)_ = 0.673, *p* = 0.76; Figure 4A], but a significant interaction in Tmx-Treated mice, at both 30 days [*F*_(11, 132)_ = 2.629, *p* = 0.005 Figure 4B1] and 146 days post-Tmx [*F*_(11, 132)_ = 2.215, *p* = 0.02; Figure 4B2]. To compare the amphetamine effect between No-Tmx and Tmx-Treated-30 d mice directly, we analyzed total locomotor activity in the hour after amphetamine administration. There was a robust genotype × treatment interaction [factorial ANOVA, *F*_(1, 24)_ = 6.436, *p* = 0.018; Figure 4C1, right]. Among treatment groups there was a significant effect of genotype in the Tmx-treated-30 d mice [*F*_(1, 12)_ = 35.916, *p* < 0.0001] but no genotype effect in the No-Tmx mice [*F*_(1, 12)_ = 0.07, *p* = 0.93]. Among genotypes, there was a treatment effect for the GLS1 iHET mice [*F*_(1, 15)_ = 5.59, *p* = 0.03], but not for the Controls [*F*_(1, 9)_ = 1.81, *p* = 0.21]. Thus, adult induction of GLS1 deficiency blocked amphetamine-induced hyperlocomotion. The attenuated locomotor response to amphetamine persisted for several months (Figure 4C1, left). Analysis of total locomotion in response to amphetamine with time (30 vs. 146 days) as the repeated measures factor revealed a genotype effect [*F*_(1, 12)_ = 23.9, *p* = 0.0003], but no time effect [*F*_(1, 12)_ = 0.91, *p* = 0.36] or time x genotype interaction [*F*_(1, 12)_ = 2.14, *p* = 0.17].

To address whether the blunted locomotor response to amphetamine in GLS1 iHET mice could have arisen as a result of increased sensitivity to psychostimulants manifest as an induction of stereotyped behaviors, we analyzed rearing and fine movements in the Tmx-treated mice. We found that total rearing counts following amphetamine did not differ between genotypes [*F*_(1, 12)_ = 3.20, *p* = 0.09], while total fine movements were lower in the GLS1 iHETs [*F*_(1, 12)_ = 16.76, *p* = 0.01]. Since there was no increase in rearing or fine movements in the GLS1 iHET mice, Tmx-treated GLS1 iHET mice were indeed less sensitive to amphetamine, and that this was not due to induction of stereotyped behaviors.

We compared the relative amphetamine-induced hyperlocomotion of Tmx-Treated-30 d and Tmx-Treated-146 d mice (Figure 4C2). Hyperlocomotion was calculated by integrating total locomotion after subtracting the average baseline locomotion in the 20 min preceding the amphetamine injection. This revealed that Tmx-treated-30 d GLS1 iHET mice showed only 5% hyperlocomotion relative to Control mice. When tested again after 5 months, Tmx-Treated-146 d GLS1 iHET showed 25% hyperlocomotion; this increase was not significant [ANOVA, with time as a repeated measures factor, *F*_(1, 8)_ = 1.1, *p* = 0.32]. Thus, the induced GLS1 allelic reduction had a persistent effect on amphetamine-induced hyperlocomotion, commensurate with chronic inhibition pharmacotherapy.

## Discussion

Evaluation of brain targets for the pharmacotherapy of neuropsychiatric disorders has been hampered by the lack of safe, specific, high-affinity, brain-penetrant inhibitors. Glutaminase is one such target, where strong evidence suggests that heterozygous reduction in glutaminase activity confers a schizophrenia resilience phenotype in mice, while at the same time having a benign side effect profile (Gaisler-Salomon et al., 2009a). Here we sought to use genetic pharmacotherapy to test whether glutaminase inhibition therapy would block propsychotic effects of amphetamine, a dimension of the schizophrenia resilience profile of GLS1 Het mice. We generated floxGLS1 mice to enable conditional genetic reduction of glutaminase expression in adulthood, and thus to reduce glutaminase activity to the level in constitutive GLS1 Het mice (Masson et al., 2006; Gaisler-Salomon et al., 2009a). We bred floxGLS1 mice with global inducible cre-deletor mice to generate GLS1 iHET mice, in which Tmx becomes an irreversible glutaminase inhibitor, and measured amphetamine-induced hyperlocomotion as a proxy for positive symptoms of schizophrenia (Arguello and Gogos, 2006; van Den Buuse, 2010). In Tmx-treated GLS1 iHET mice, amphetamine-induced hyperlocomotion was blocked, consistent with the therapeutic potential of glutaminase inhibition.

### Generation of floxGLS1 mice

We made conditional GLS1 mice by floxing the GLS1 coding sequence in GLS1 exon 1. GLS1 mRNA levels were unaffected in heterozygous, as well as homozygous floxGLS1 mice indicating that the floxGLS1 allele is a silent mutation. In contrast, GLS1 knockout mice die shortly after birth due to respiratory failure, apparently the result of altered patterns of respiratory rhythms in brainstem respiratory centers (Masson et al., 2006). In a preliminary experiment, we have found that inducible GLS1 knockout mice, GLS1 iHOM (CAG ^ERT2*cre*∕+^:: GLS1^*lox*∕*lox*^) mice (Control, *n* = 2; Tmx-treated, *n* = 2) develop a severe neurological syndrome about 3 weeks post-Tmx, revealing the necessity of glutaminase function in adulthood. This observation further indicated that cre-mediated recombination of the floxGLS1allele produced a delta (knockout) allele, and that 1 month post-Tmx was sufficient for induction of glutaminase deficiency. While this project involved generating a floxGLS1 mouse line, this first step in the implementation of a genetic pharmacotherapy strategy for other CNS targets is now increasingly facile, as floxed mouse lines have been generated for the majority of mouse genes through the Knockout Mouse Project (www.komp.org), including for GLS1 (Skarnes et al., 2011), and floxed mouse lines can be rapidly generated using CRISPR-Cas genome engineering (Yang et al., 2014).

### Validation of GLS1 iHET mice

GLS1 iHET mice showed full recombination following Tmx activation of CAG^ERT2*cre*^, as evidenced by complete loss of the floxed GLS1 allele. However, they showed significant leakiness, with about 50% recombination in the absence of Tmx, and a 25% reduction in functional allelic abundance. At the point of Tmx treatment in adulthood, this leakiness had not impacted mRNA levels; post-Tmx, mRNA levels went down to about 50%. Constitutive GLS1 HET mice show a similar 50% reduction in mRNA (Gaisler-Salomon et al., 2012), corresponding to a >50% reduction in glutaminase activity (Gaisler-Salomon et al., 2009a). However, *ex vivo* measurement of glutaminase activity does not reflect *in vivo* glutaminase activity, which is subject to multimodal modulation due to end-product inhibition, substrate availability, and phosphate modulation (Curthoys and Watford, 1995). Rather, *ex vivo* activity reflects the amount of glutaminase protein present, reported by its maximal activity. Evaluating glutaminase inhibition *in vivo* requires tracking ^13^C-precursor flux through astrocytes to neurons and the conversion of glutamine to glutamate by magnetic resonance spectroscopy (Patel et al., 2010; El Hage et al., 2012).

To generate a genetic intervention that mimics a pharmacological effect, our strategy requires cre activation with a ubiquitous promoter that only produces recombination after Tmx administration. The progressive recombination due to leakiness found in this study limits the tractability of a strategy with CAG^ERT2*cre*^ mice, and highlights the importance of measuring recombination prior to Tmx administration when using inducible mouse lines. Other ubiquitous inducible promoters should be essayed, such as ROSA^ERT2*cre*^ (Ventura et al., 2007) and UBC^ERT2*cre*^ (Ruzankina et al., 2007). However, these promoters are weaker and may not achieve full recombination of some floxed alleles. Breeding mice homozygous for the ERT2*cre* allele would be one way to increase the efficacy of weaker promoters. Another way would be improved delivery of Tmx by solubilization of Tmx with hydroxybutenyl-cyclodextrin (Buchanan et al., 2006).

### Testing whether induction of glutaminase deficiency is potentially therapeutic

To test the efficacy of adult-onset glutaminase inhibition, we chose a key dopamine-dependent behavioral phenotype of constitutive GLS1 HETs, the attenuated response to pro-psychotic amphetamine challenge (Gaisler-Salomon et al., 2009a). Amphetamine-induced hyperlocomotion is a robust, efficient test primarily involving dopaminergic and not glutamatergic mechanisms. Repeated amphetamine produces sensitization (Vezina, 2004), so the attenuation of amphetamine-induced hyperlocomotion we found on the tests done post-Tmx went counter to the expected increase with repeated administration. Amphetamine-induced hyperlocomotion, driven by increased dopamine release in the NAc (Sellings and Clarke, 2003; Ikemoto, 2007), along with PPI (Powell et al., 2009), which was unaffected in constitutive GLS1 HETs (Gaisler-Salomon et al., 2009a), are the two tests with the best face validity for positive symptoms in SCZ (Arguello and Gogos, 2006; van Den Buuse, 2010).

The low-dose Tmx dose regimen used (1 mg daily for 5 days) has no persistent impact on a range of mouse behaviors, examined 1 month post-Tmx (Vogt et al., 2008), and did not attenuate amphetamine-induced locomotion, as seen in control mice at the two post-Tmx time points tested. Low-dose Tmx with a heterozygous CAT-ERT2*cre* allele should similarly limit ERT2*cre* activation and minimize toxicity due to recombination at pseudo-loxP sites (Higashi et al., 2009). The absence of body weight differences between Control and GLS1 iHET mice indicates that ERT2*cre* activation in the GLS1 iHETs did not produce significant toxicity and *per se* did not reduce the response to amphetamine. Having now shown that the floxGLS1 allele is a silent mutation that does not impact gene expression, further studies would be better controlled by varying flox status. Evidently, limiting Tmx dosage to levels that would induce efficient recombination of the gene of interest without inducing significant Tmx or ERT2*cre* toxicity, and allowing a month for recovery, will be crucial for successful implementation of genetic pharmacotherapy.

**Figure 5:**
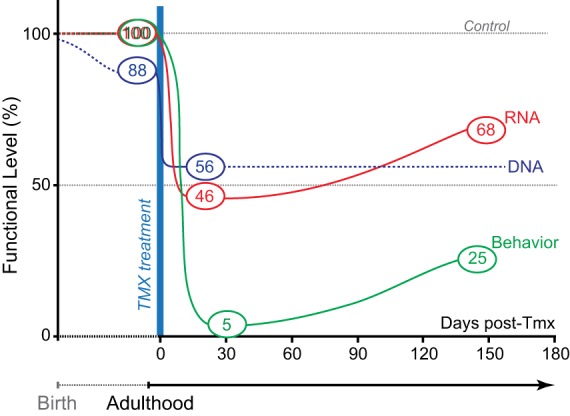
**Genetic pharmacotherapy for glutaminase inhibition**. The relative timing and magnitude of DNA, RNA, and behavioral effects are schematized prior to and after Tmx treatment. Black dotted line shows extrapolated DNA levels. Prior to Tmx, there was significant leaky recombination, but this did not impact GLS1 mRNA levels or behavior (amphetamine-induced locomotion). After Tmx, there was full recombination. At short and long-time points post-Tmx, both mRNA levels and amphetamine-induced hyperlocomotion showed the maximum effect at the short-time point. As in constitutive GLS1 Hets, amphetamine-induced hyperlocomotion was blocked at the short-time point, and strongly attenuated at the long-time point.

With Tmx treatment, GLS1 mRNA levels in GLS1 iHET mice went from normal to heterozygous levels. Prior to Tmx treatment, Control and GLS1 iHET mice showed identical responses to amphetamine. After Tmx treatment, and a sufficient time interval to allow the impact of the allelic reduction to translate into reduced mRNA levels and presumed protein levels, there was a marked reduction in amphetamine-induced hyperlocomotion (see Figure 5). This was the same marked reduction we had seen in constitutive GLS1 HETs (Gaisler-Salomon et al., 2009a), indicating that reduction of GLS1 to heterozygous levels in adulthood, was sufficient to reduce amphetamine responsivity.

GLS1 iHOM mice could be used to access the whole dose-response range by varying Tmx dose and the frequency of administration to induce varying degrees of recombination, extending to complete inhibition (as noted above this would include lethality with full inhibition). However, the degree of recombination, determined by allelic abundance, would need to be assessed for every mouse, so larger cohorts would be required to obtain sufficient numbers in the desired dose range. Moreover, achieving a given inhibition in GLS1 iHOM mice would be fraught with variability in the degree of recombination, not only between animals, but also between different cell populations in the same animal. So, utilizing GLS1 iHET mice, as we did here, is the better strategy as it enables end-stopping glutaminase inhibition to heterozygous levels.

### Genetic pharmacotherapy as a strategy for psychiatric drug development

Genetic intervention to achieve a pharmacological response, which we term *Genetic Pharmacotherapy* (Gellman et al., 2011), has been used widely outside the nervous system to modulate pathogenic pathways and demonstrate therapeutic efficacy, particularly in cancer research (Ruzankina et al., 2007; Ventura et al., 2007; Higashi et al., 2009; Herranz et al., 2015), but has not been used previously for CNS disorders. The present study highlights the advantages of the approach for neuropsychiatric drug development. Targets can be tested in advance of formulating specific, brain-penetrant inhibitors. The genetic intervention achieves perfect target specificity, with the only off-target effects being those of Tmx, which are controlled for by treating both control and genetically modified mice. The present genetic pharmacotherapy study establishes the viability of the strategy to identify new druggable targets for the treatment of psychiatric disorders. Our initial evaluation suggests that investment in molecular entities that inhibit glutaminase could prove therapeutically effective. Further studies should address other dimensions of the SCZ-resilience phenotype, and confirm the benign side-effect profile of adult-induced global glutaminase reduction.

Our results align with the finding that the recently-recognized glutaminase inhibitor ebselen (Thomas et al., 2013) attenuates amphetamine-induced hyperlocomotion (Singh et al., 2013). Ebselen has also been shown to rescue prepulse inhibition deficits in the neonatal ventral hippocampal lesion (NVHL) rodent schizophrenia model (Cabungcal et al., 2014). In both these reports, the putative mechanism of action involves ebselen acting as a glutathione peroxidase mimic to reduce oxidative stress (Sies, 1993). However, *in vitro* ebselen inhibits glutaminase activity with an K_*i*_ of 0.015 μM (Thomas et al., 2013), which is almost 3 orders of magnitude better than the recognized glutaminase inhibitor BPTES with an IC_50_ of 3 μM (Thomas et al., 2013) or the bromo-benzophenanthridinone compound 968, also with an IC_50_ in the low μM range (Wang et al., 2010), arguing that its behavioral effects are mediated via glutaminase inhibition. Thus, the ebselen data provide further support for the therapeutic potential of glutaminase inhibition for schizophrenia, and suggest that ebselen may have therapeutic potential in the treatment of the disorder.

Genetic pharmacotherapy could be used to verify the targets of known psychotherapeutic drugs though common actions on phenotypic markers. The strategy tests chronic treatment, as we have shown in the persistence of the behavioral effects in GLS1 iHETs months after Tmx treatment. In the case of the putative action of ebselen as a glutaminase inhibitor, the action of the drug could be compared to Tmx treatment of GLS1 iHET mice, and the biomarker of glutamine flux through glutaminase measured with ^13^C-MRS. Genetic pharmacotherapy could be applied to mouse models with construct validity, such as mice carrying a disease mutation, or models generated through developmental or environmental interventions to reverse pathological phenotypes. However, before the approach can be used effectively, global-inducible promoters must be identified that show minimal leakiness and yet achieve full cre-mediated recombination.

The strategy outlined here may be seen as a means to stimulate serendipitous discovery of novel psychotropics—as a genetically engendered resilience phenotype can be tested with genetic pharmacotherapy to determine whether inhibition is potentially therapeutic. The dearth of new psychiatric drugs over the past decades underpins the vastly unmet need for better treatments (Brundtland, 2001; Miller, 2010). Critics of psychiatric drug development argue that because the etiology of major mental illnesses remains so poorly understood, adequate treatments cannot be developed (Conn and Roth, 2008). Using genetic pharmacotherapy to test mouse models of disease resilience promises to make a larger molecular landscape accessible to drug discovery and enable more rapid testing of psychopharmacotherapeutic targets.

## Author contributions

SM, JM, IG, RH and SR conceptualized the research; JM and RH developed the GLS1 targeting strategy; JM, GT, CL, RM and IG made the floxGLS1 mice; SM, CG, YW and RE determined allelic abundance and gene expression; SM and CG conducted the behavioral studies; SM, CG and SR wrote the manuscript.

## Funding

This work was supported by NIH K02 DA000356, R21 DA014055, P50 MH066171, R01 DA017978, R01 MH087758, and a Columbia University Research Initiatives in Science & Engineering (RISE) award (SR); the Frontier Fund of the Columbia Department of Psychiatry and the Philippe Foundation (JM); NARSAD (SR, JM); T32 DA016224 and the Rothschild Foundation (IG); and P30 CA013696 (Transgenic Mouse Facility of the Herbert Irving Comprehensive Cancer Center, Columbia University).

### Conflict of interest statement

The authors declare that the research was conducted in the absence of any commercial or financial relationships that could be construed as a potential conflict of interest. René Hen receives compensation as a consultant for Lundbeck, Servier, and Roche. All other authors report no biomedical financial interests or potential conflicts of interest.
